# Correlation between the AMADEUS score and preoperative clinical patient-reported outcome measurements (PROMs) in patients undergoing matrix-induced autologous chondrocyte implantation (MACI)

**DOI:** 10.1186/s13018-019-1107-z

**Published:** 2019-03-22

**Authors:** Armin Runer, Pia Jungmann, Götz Welsch, Danica Kümmel, Franco Impellizzieri, Stefan Preiss, Gian Salzmann

**Affiliations:** 1 Department for Trauma Surgery, Medical University of Innsbruck, Anichstrasse 35, 6020 Innsbruck, Austria; 2 Schulthess Clinic Zurich, Zurich, Switzerland; 3 Department of Neuroradiology, University Hospital Zurich, University of Zurich, Zurich, Switzerland; 4 Athleticum, University Hospital of Hamburg-Eppendorf, Hamburg, Germany

**Keywords:** AMADEUS score, Matrix-induced autologous chondrocyte implantation, MACI, Chondral lesions, Patient-reported outcome measures

## Abstract

**Background:**

Recently, the AMADEUS (Area Measurement And DEpth Underlying Structures) grading system has been introduced to evaluate and grade osteochondral lesions prior to cartilage surgery. The AMADEUS score has not been connected to clinical data in order to test a potential clincial impact.

**Purpose:**

To examine the correlation between the AMADEUS score and preoperative patient-reported outcome measurements (PROMs).

**Study design:**

Case series

**Methods:**

Patients treated with matrix-induced autologous chondrocyte implantation (MACI) were included in the study, unless exclusion criteria like BMI > 35, prior extensive meniscectomy or ongoing inflammatory arthritis were present. Preoperative magnetic resonance (MR) examinations were graded according to the standardized AMADEUS protocol. The final AMADEUS score was correlated with preoperative patient-reported outcome measurements (PROMs), including the IKDC (International Knee Documentation Committee), the Lysholm score, the Short-Form-12 (SF-12) score, and the Core Outcome Measures Index (COMI) score.

**Results:**

A total of 50 patients with a mean age of 33.6 ± 11.5 years, a mean BMI of 25.1 ± 4.9, and a mean defect size of 2.3 ± 1.5 cm^2^ were included in the study. More severe cartilage defects, indicated by the AMADEUS grade (*R* = 0.35, *p* = 0.01) and the AMADEUS score (*R* = − 0.36, *p* = 0.01) as well as larger chondral defects (*R* = 0.32, *p* = 0.03) show a moderate correlation with the higher COMI scores. No correlative capacity was demonstrated for the AMADEUS score and the IKDC, Lysholm, and Tegner activity scores as well as for its subscales.

**Conclusion:**

There is a moderate correlation of the COMI and the AMADEUS score in patients treated with matrix-induced autologous chondrocyte implantation (MACI). All other patient-reported outcome measurement scores (PROMs) show no evidence of an association to the magnetic resonance-based AMADEUS score.

**Clinical relevance:**

The clinical and scientific implication of the COMI score as a PROM tool can be recommended when working with the AMADEUS score and patients undergoing MACI.

## What is known about the subject

There is an increased interest in Matrix-induced autologous chondrocyte implantation (MACI) as a method to treat patients with condral defects. Preoperatively reliable radiological classification and outcome tools for chondral lesions like the recently developed AMADEUS score are needed and requested. So far, the AMADEUS has not been connected to clinical data in order to test a potential clinical impact.

## What this study adds to existing knowledge

There is a moderate correlation between the COMI score and the preoperative AMADEUS grade, AMADEUS total score, AMADEUS area size, and calculated chondral defect size. However, limited correlative capacity was demonstrated between other frequently used PROMs.

## Introduction

Matrix-induced autologous chondrocyte implantation (MACI) has become an important and widely used treatment option for large, full-thickness chondral defects of the knee [1, 2]. This has created the need and desire for reliable radiological classification and outcome tools for chondral lesions both pre- and postoperatively.

Several classification systems such as the Outerbridge scoring system or the ICRS (International Cartilage Repair Society) scoring systems exist, each with specific advantages and disadvantages [3, 4].

Recently, the AMADEUS (Area Measurement And DEpth Underlying Structures) grading system has been introduced. This magnetic resonance (MR)-based classification system was developed in order to evaluate and grade osteochondral lesions prior to cartilage surgery [5]. The AMADEUS score is a three-part classification system rating cartilage defect size, depth, and subchondral bone of the defect providing a three-digit code for each part based on the various defect characteristics. Ultimately, an overall AMADEUS score (0 = worst score, 100 = no chondral defect) and a final AMADEUS grade (grade I = best, grade IV = worst) are provided. The AMADEUS score was developed in order to facilitate therapeutic and surgical decision-making, and interdisciplinary and patient communication as well as multicenter comparison [5]. So far, the AMADEUS has not been connected to clinical data in order to test a potential clinical impact. The aim of the present study was to examine the correlation between the AMADEUS grading system and frequently used, preoperative patient-reported outcome measurements (PROMs) like the IKDC (International Knee Documentation Committee), the Lysholm score, the Tegner activity score, and the Core Outcome Measures Index (COMI) score.

## Methods

The study was conducted according to the Declaration of Helsinki (World Medical Association) and approved by the Kantonalen Ethikkommision Zürich (PB_2017-00307). From all patients, written and verbal informed consent was obtained prior to study inclusion.

Patients treated with MACI between October 2015 and December 2016 were included in the study. Exclusion criteria were a BMI > 35, prior extensive meniscectomy, ongoing progressive inflammatory arthritis, or previous ligamentous injury. All surgical interventions were performed by the senior author (GS). Indication, execution, and rehabilitation for MACI were according to standard guidelines [6].

Each patient received standard preoperative 3-T or 1.5-T MR examination with sequences including two-dimensional (2D) intermediate-weighted (IM-w) turbo spin echo (TSE) images in at least two planes and a T1-w TSE sequence in at least one plane (sagittal or coronal) [5]. Imaging parameters were used in accordance to Jungmann et al. [5]. MR images were transferred on a picture archiving and communication system (PACS) workstation (Easy Vision, Philips, Best, Netherlands) and were graded according to the AMADEUS grading system. In addition, different patient-administered outcome scores were obtained.

### AMADEUS grading

AMADEUS grading was performed according to the standard AMADEUS grading protocol previously described in detail by Jungmann et al. [5] by one experienced orthopedic surgeon (AR) who was not involved in the clinical setting. Briefly, the cartilage defect area was calculated by measuring the defect diameter in two planes. Transverse and sagittal images were used measurements of defects located at the patella, whereas sagittal and coronal images were used measurements of defects located at the femur or at the tibia. Defect depth was graded on IM-w images and classified according to the most severe condition of the defect as “severe signal alteration (a),” “partial thickness defects (b),” “full thickness (c),” or “no defect”. Underlying structures were classified as (A) if the subchondral lamina was intact and no morphological defect of the subchondral bone was visible. Subchondral bony defects and/or any other subchondral pathologies (ganglia, cysts, necrotic tissue) of less than 5-mm depth were graded as (B). Defects of 5-mm depth or more that required surgical repair were graded as (C). In addition to the grading of the defect depth, the presence of bone-marrow edema was graded as (E). AMADEUS score is the sum of the corresponding subscores, ranging from 100 (= no osteochondral defect) to 0 (= severe cartilage defect). Based on the total AMADEUS score, an AMADEUS grade was assigned to each patient giving an overall estimate of the lesion: grade I, score > 75; grade II, score > 50 and ≤ 75; grade III, score > 25 and ≤ 50; and grade IV, score ≤ 25, grade I being the least severe defect and grade IV being the most severe defect.

### Patient-reported outcome measures (PROM)

On surgery admission day, every patient completed four patient-administered outcome scores including the IKDC (International Knee Documentation Committee), the Lysholm score, the Tegner activity score, and the Core Outcome Measures Index (COMI) score. The IKDC score is a frequently used, knee-specific questionnaire including 18 questions focusing on symptoms, sports, and daily activity as well as current knee function [7]. The Lysholm score, designed to evaluate knee function and pain, includes the grading of the following eight items: limp, support, locking, instability, pain, swelling, stair climbing, and squatting [7]. The Tegner activity score was developed to complement the Lysholm score. It provides a standardized method of grading work and sport activities [7]. The COMI score, originally designed for spine and later adapted for knee patients, is a single set of six items assessing pain, function, quality of life, and disability in patients undergoing knee surgery. A lower score represents hereby a better overall knee situation [8].

### Surgical technique

A standard, two-stage surgical MACI technique was used as previously described in detail [9–12]. In short, diagnosis and surgery indication was confirmed by routine arthroscopy. Subsequently, two osteochondral cylinders were harvested from a non- or low-weight-baring area of the intercondylar notch, and cell expansion and chondrocyte seeding were initiated. After 4 to 6 weeks, standardized MACI implantation was performed using open mini knee arthrotomy. Coordinated rehabilitation program, including continuous passive motion (CPM) and limited weight bearing for at least 6 weeks, was initiated after the first postoperative day.

### Statistics

Statistical analysis was performed using SPSS v.20 (IBM Corp.). Patient demographics and chondral defect characteristics were calculated using means and standard deviation (SD). Normal distribution was tested using the Kolmogorov-Smirnov test. Strength and association between radiological data and PROMs was calculated applying the nonparametric Spearman’s rank correlation coefficient (SCC). A one-way ANOVA with Bonferroni post hoc test was used to determine differences between the means of two or more independent groups.

## Results

A total of 50 patients, 31 males and 19 females, were included in the study. Detailed patient characteristics are displayed in Table 1.

**Table 1:** Patient demographics

Gender	*N*	Age (years)	Height (cm)	Weight (kg)	BMI	Existing pain (month)	Smoking		Painkillers	
							Yes/no	%	Yes/no	%
Male	31	37.1 (± 11.6)	178.8 (± 5.7)	81.9 (± 15.8)	25.7 (± 5.0)	24.7 (± 27.7)	8/23	26/74	7/24	23/77
Female	19	28.0 (± 9.2)	168.4 (± 7.5)	68.7 (± 14.9)	24.2 (± 4.6)	39.5 (± 58.2)	6/13	32/68	12/7	62/38
Total	50	33.6 (± 11.5)	174.8 (± 8.2)	76.9 (± 16.6)	25.1 (± 4.9)	30.3 (± 42.0)	14/26	28/72	19/31	38/62

Values are reported in mean (± standard deviation). Mean and standard deviation (SD) for the chondral defect size area and patient-reported outcome measures (PROM) in respect to the different AMADEUS grades

Detailed measured diameters and calculated chondral defect size of the cohort are given in Table 2. Thirty-six percent (*n* = 18) of the chondral lesions were located at the retropatellar surface, 32% (*n* = 16) at the medial femoral epicondyle, 18% (*n* = 9) at the lateral femoral epicondyle, 10% (*n* = 5) in the trochlear groove, 2% (*n* = 1) at both the medial and lateral femoral epicondyle, and 2% (*n* = 1) at the tibial plateau.

**Table 2:** Diameters and area defects of chondral lesions displayed by mean, standard deviation (SD), minimum, and maximum

	Diameter D1 (mm)	Diameter D2 (mm)	Area of defect (mm^2^)
Mean	16.20	13.80	2.30
SD	5.50	5.50	1.50
Minimum	7.20	3.30	0.50
Maximum	27.60	31.70	7.50

*SD* standard deviation

The AMADEUS subscores, mean AMADEUS score, AMADEUS gradings, and core grading are provided in Table 3.

**Table 3:** Detailed grading of the AMADEUS score of all included patients

AMADEUS feature	Score	Frequency	Percent
Area measurement			
Defect size			
No defect	40	0	0
≤ 1 cm^2^	35	8	16
> 1 to ≤ 2 cm^2^	30	18	36
> 2 to ≤ 4 cm^2^	20	21	42
> 4 to	10	1	2
≤ 6 cm^2^	0	2	4
Defect depth			
(n) No defect	20	0	0
(a) Signal alteration	15	2	4
(b) Partial-thickness defect	10	20	40
(c) Full-thickness defect	0	28	56
Underlying structures			
Subchondral bone defect			
A. no defect	30	35	70
B. bony defect/cyst ≤ 5-mm depth	20	6	12
C. bony defect/cyst > 5-mm depth	0	9	18
Addendum—potential fourth digit			
No defect-associated BME	10	27	54
E. defect-associated BME	0	23	46
AMADEUS total score	100	Mean 58.4 (± 20.4)	
AMADEUS grade	(0 worst, 100 best)		
Grade I	> 75	9	18
Grade II	> 50 and ≤ 75	25	50
Grade III	> 25 and ≤ 50	11	22
Grade IV	≤25	5	10

*BME* bone marrow edema-like lesion, *BMI* body mass index, *AMADEUS* Area Measurement And DEpth Underlying Structures

The correlation of the different PROMs (COMI, IKDC, Lysholm score, Tegner score) with the AMADEUS grade, the AMADEUS score, and the four AMADEUS subscores is displayed in Table 4.

**Table 4:** Correlation of the different AMADEUS score items in regard to the patient-reported outcome measures (PROM). The COMI score is the only PROM score showing significant correlation to subscales of the radiological AMADEUS score

	AMADEUS grade		AMADEUS total score		Defect size		Area score		Defect depth score		Underlying structure score		Addendum score	
	Sp. *R*	*P*	Sp. *R*	*P*	Sp. *R*	*P*	Sp. *R*	*P*	Sp. *R*	*P*	Sp. *R*	*P*	Sp. *R*	*P*
COMI score	0.35	0.01	− 0.36	0.01	0.32	0.03	− 0.32	0.02	− 0.23	0.10	− 0.22	0.12	− 0.18	0.22
IKDC score	− 0.18	0.22	0.16	0.26	− 0.14	0.32	0.11	0.44	0.18	0.21	0.03	0.86	0.00	1.00
Lysholm score	− 0.14	0.32	0.14	0.32	− 0.15	0.30	0.08	0.61	0.11	0.45	0.02	0.89	0.83	0.56
Tegner score	− 0.13	0.38	0.12	0.42	0.03	0.85	0.03	0.84	0.27	0.05	− 0.06	0.68	0.04	0.80

*Sp. R* Spearman *R*

Statistical analysis comparing defect size and PROMs within the different grades of the AMADEUS grade (grade I–IV) showed a statistically significant difference for “Defect size” (*p* < 0.01). The post hoc test revealed a statistically significant increase in the chondral defect area between the AMADEUS grade I and grade III (*p* ≤ 0.01) as well as between grade I and grade IV (*p* < 0.01). No significant difference was found between the final values of the different PROMs (Table 5).

**Table 5:** Mean and standard deviation (SD) for the chondral defect size area and patient-reported outcome measures (PROMs) in respect to the different AMADEUS grades

AMADEUS Grade	Defect size (cm^2^)		COMI score		IKDC score		Lysholm score		Tegner score	
	Mean	SD	Mean	SD	Mean	SD	Mean	SD	Mean	SD
1	1.31	0.50	5.29	1.58	52.62	16.10	58.33	17.73	3.89	2.421
2	1.95	0.96	5.23	2.13	49.01	14.87	54.88	20.79	2.72	1.595
3	3.12	1.58	6.13	1.27	44.83	13.52	54.00	19.68	2.45	1.128
4	4.55	2.01	6.95	1.38	43.20	16.29	45.00	22.03	3.80	3.834
Sig.	< 0.01		0.19		0.58		0.69		0.27	

*SD* standard deviation

**Fig. 1:**
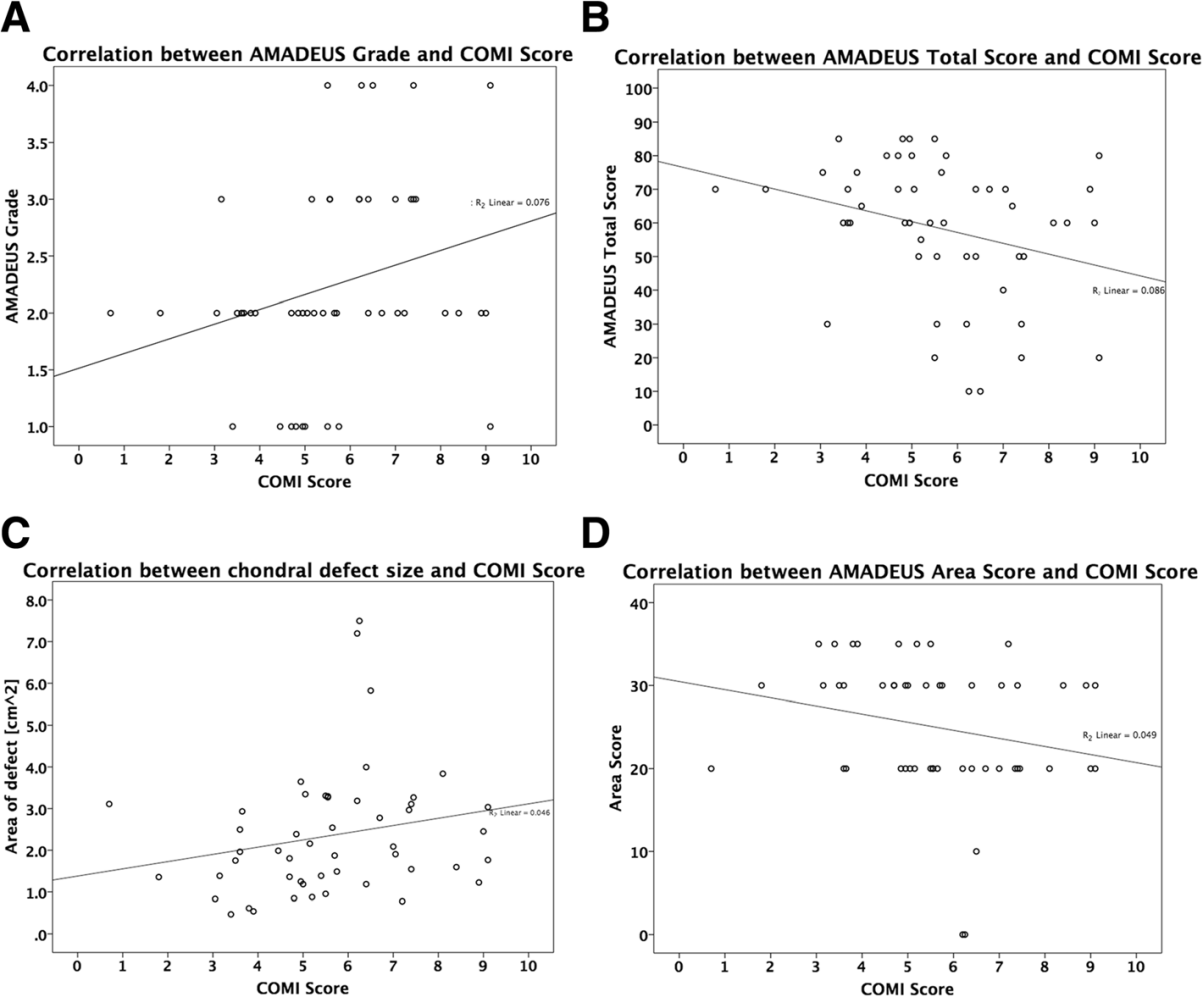
Scatter graphs showing the correlation between COMI score and AMADEUS grade (**a**), AMADEUS total score (**b**), chondral defect size (**c**), and AMADEUS area score (**d**). A lower clinical COMI score positively correlates with the radiological AMADEUS grade (**a**) as well as the chondral defect size (**b**), representing an overall better knee situation. The COMI score negatively correlates with the AMADEUS total score (**b**) and the AMADEUS area score (**d**), indicating a worse knee situation

**Fig. 2:**
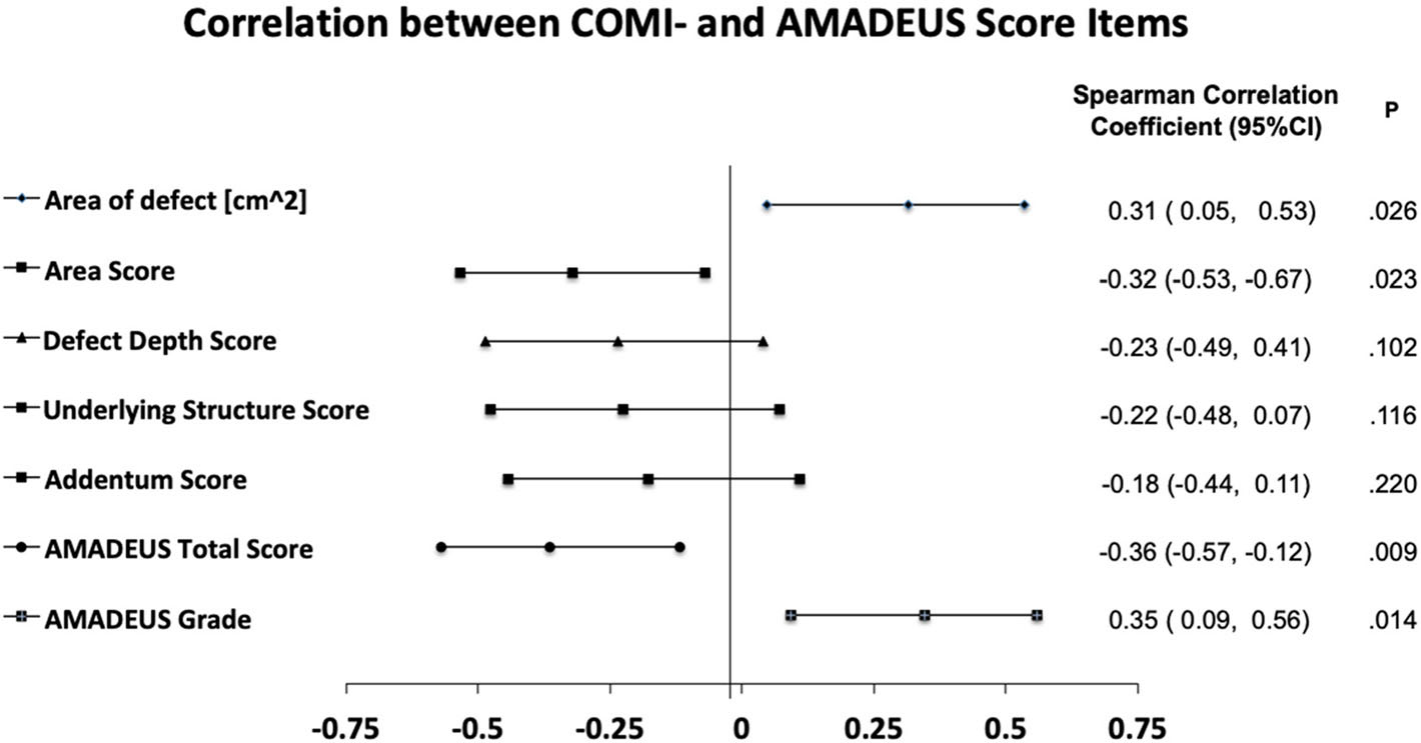
Forest plot showing the correlation coefficient, *P* value, and 95% confidence interval (95% CIs) between the COMI score and the MRI-based subscales of the AMADEUS score as well as the AMADEUS total score and AMADEUS grade. A significant positive correlation exists between the AREA of defect [cm^2^], the AMADEUS grade, and the COMI score. A greater chondral defect and a higher AMADEUS grade results therefore in a higher COMI score representing an overall worse knee situation. A significant negative correlation is shown between the AREA score, the AMADEUS total score, and the COMI score. A lower AREA score and lower AMADEUS total score results therefore in a higher COMI score indicating a lower overall knee situation

More severe cartilage defects as indicated by the AMADEUS grade (*R* = 0.35, *p* = 0.01) and the AMADEUS total score (*R* = − 0.36, *p* = 0.01) as well as larger chondral defects *R* = 0.32, *p* = 0.03) represented by a lower “area score” (*R* = − 0.32, *p* = 0.02) show moderate correlative capacity with higher COMI scores (Fig. 1). Figure 2 shows the SCC with P-values and 95%-CIs for each of the AMADEUS items, the AMADEUS overall score, the AMADEUS Grade and the COMI Score. No statistically significant correlation was shown between the COMI score and the “defect depth score,” the “underlying structure score,” or the “addendum score.” The IKDC, Lysholm, and Tegner activity scores showed no significant correlation with the AMADEUS score or its subitems.

## Discussion

The purpose of this study was to evaluate the correlation between the AMADEUS score and frequently used PROMs in patients undergoing MACI in order to test a potential clinical impact. The results demonstrate a moderate correlation between the COMI score and the preoperative AMADEUS grade, AMADEUS total score, AMADEUS area size, and defect size. No correlative capacity was demonstrated for the AMADEUS score and the IKDC, Lysholm, and Tegner activity scores as well as for its subscales. This study is the first attempt to correlate the AMADEUS score and its subscales to frequently used PROMs.

While there is abundant literature focusing on the correlation of postoperative radiological and postoperative clinical data in patients after MACI surgery, little is known about the relationship between preoperative radiological grading and preoperative clinical status before MACI surgery. Previous studies correlating preoperative radiological and clinical data were focused on knee osteoarthritis prior to total knee replacement. Larsson et al. [13] showed limited correlations between knee pain, radiographic osteoarthritis (OA), and functional capacity. In a systematic review focusing on patient with OA, Bedson and Croft [14] concluded that radiographs are not very precise in predicting knee pain or disability. Hernandez-Vaquero and Fernandez-Carreira [15] found no correlation between the Ahlbäck classification for OA and the subjectively reported “quality of life”. According to Bedson and Croft [14] there might be several reasons why such a discordance of radiological and clinical data may arise: (1) by not taking all possible X-ray views evaluation, the true radiographic prevalence of a disease might be underestimated, (2) the definition of pain and the grading of radiographic severity have a strong influence on the correlation between radiographic and clinical data, and (3) the study population with respect to age, ethnicity, or activity level has an influence on the relationship between clinical and radiological data.

Furthermore, a variety of different factors, such as the social environment, suboptimal communication between patient and physician, or the assessment of pain, may influence the outcome of PROMs [15].

The AMADEUS score was designed and recently introduced in order to provide a preoperative overview and grading of osteochondral knee lesions in patients undergoing possible surgery [5]. Based on MRI imaging, it uses the three most important components of osteochondral defects (size, depth, and subchondral bone) to provide patients, radiologists, researcher, and surgeons an overview of the knee chondral situation and provide a rationale for treatment strategies and decisions [5]. Furthermore, the AMADEUS score represents a preoperative equivalent to the widely used MOCART score, which is used for postoperative assessment of the cartilage tissue quality and repair [16]. Therefore, the AMADEUS score can be used to provide an extensive and comparable picture of the patient’s preoperative chondral situation on one hand, and on the other hand, it provides reliable baseline data allowing comparisons of the preoperative to postoperative findings.

Several reasons might explain the rather weak correlative capacity of clinical outcomes with the AMADEUS score. First, the large number of variables, of which composite scores like the MOCART or AMADEUS score are composed, may influence the association with clinical scores [2]. Second, other factors that were not included in these scores can influence the clinical outcome, e.g., inflammation, increased vascular penetration, or nerve growth [2]. Moreover, patient-specific parameters including age, BMI, nicotine abuse, previous surgical treatments, duration of symptoms, the applied postoperative rehabilitation protocol, patient expectation, and its individual pain perception as well as defect-specific parameters like defect location, age of the defect, containment, and number of defects have an influence on the clinical and functional outcome but are not measured and considered in radiological scores [17–19].

The outcomes of the present work are in line with the results of previously conducted correlative studies by showing only limited and weak correlation of radiological and clinical data [20–23]. However, a positive association to the recently introduced COMI score was shown. Therefore, the clinical and scientific implication of the COMI as a PROMs tool is recommended when working with the AMADEUS score and patients undergoing MACI.

This study has some strengths and limitations. It must be emphasized that the number of patients could have limited our study results and that a larger sample size would have been favorable. Despite all questionnaires used in this study were previously validated to psychometric parameters and good responsiveness, patient-reported outcome measurements (PROMs) always carry a potential bias or misunderstanding of the questions. A strength of the study was the fact that all patients were examined, operated, and followed by one highly trained and experienced surgeon only. Furthermore, the radiological AMADEUS grading was not performed by the surgeon itself but by an independent and clinical outcome-blinded researcher.

## Conclusion

In conclusion, a moderate correlation between the COMI and AMADEUS score is shown in patients treated with matrix-induced autologous chondrocyte implantation (MACI). All other patient-reported outcome measurement scores (PROMs) show no evidence of an association. The clinical and scientific implication of the COMI score as a PROM tool can be recommended when working with the AMADEUS score and patients undergoing MACI.

## Abbreviations

AMADEUS: Area Measurement And DEpth Underlying Structures
COMI: Core Outcome Measures Index score
ICRS: International Cartilage Repair Society
IKDC: International Knee Documentation Committee
MACI: Matrix-induced autologous chondrocyte implantation
MR: Magnetic resonance
OA: Osteoarthritis
PACS: Picture archiving and communication system
PROMs: Patient-reported outcome measurements
